# The role of wrist-worn technology in the management of Parkinson’s disease in daily life: A narrative review

**DOI:** 10.3389/fninf.2023.1135300

**Published:** 2023-04-12

**Authors:** Peng Li, Richard van Wezel, Fei He, Yifan Zhao, Ying Wang

**Affiliations:** ^1^Biomedical Signals and Systems (BSS) Group, Faculty of Electrical Engineering, Mathematics and Computer Science (EEMCS), University of Twente, Enschede, Netherlands; ^2^Department of Biophysics, Donders Institute for Brain, Cognition and Behaviour, Radboud University, Nijmegen, Netherlands; ^3^Centre for Computational Science and Mathematical Modelling, Coventry University, Coventry, United Kingdom; ^4^School of Aerospace, Transport and Manufacturing, Cranfield University, Cranfield, United Kingdom

**Keywords:** Parkinson’s disease, wrist-worn, sensor, daily life, monitoring, management

## Abstract

Parkinson’s disease (PD) is a neurodegenerative disorder that affects millions of people worldwide. Its slow and heterogeneous progression over time makes timely diagnosis challenging. Wrist-worn digital devices, particularly smartwatches, are currently the most popular tools in the PD research field due to their convenience for long-term daily life monitoring. While wrist-worn sensing devices have garnered significant interest, their value for daily practice is still unclear. In this narrative review, we survey demographic, clinical and technological information from 39 articles across four public databases. Wrist-worn technology mainly monitors motor symptoms and sleep disorders of patients in daily life. We find that accelerometers are the most commonly used sensors to measure the movement of people living with PD. There are few studies on monitoring the disease progression compared to symptom classification. We conclude that wrist-worn sensing technology might be useful to assist in the management of PD through an automatic assessment based on patient-provided daily living information.

## 1. Introduction

Parkinson’s disease (PD) is a rapidly growing neurological disorder that affects people worldwide, especially those over 65 years old. In the past three decades, the social burden of PD has doubled due to the ageing of the global population. If this trend continues, the predicted number of patients will exceed 12 million in the next 30 years, which will have a significant economic impact on our society (Rocca, 2018). There is an urgent need to identify new effective and affordable interventions to reduce the impact of PD on patients and society (G 2016 Parkinson’s Disease Collaborators, 2018).

Clinically, PD patients typically present motor symptoms for the first clinic visit, such as rest tremor, bradykinesia, and rigidity. Healthcare professionals can often diagnose PD when patients present these classical motor symptoms at a late stage, according to the UK Brain Bank Criteria (Calne et al., 1992). Freezing of gait (FoG), posture instability, or gait disturbances are also motor symptoms in daily life but aren’t belonging to the currently diagnosed criteria according to the UK brain bank. The Braak stages show that some prodromal non-motor symptoms (NMS), such as constipation and sleep disorder, can occur before Lewy bodies (LB) appear in the substantia nigra of the brain stem (Rietdijk et al., 2017). Early detection of prodromal parkinsonism is crucial to the effectiveness of disease-modifying interventions. Moreover, emergency measures are insufficient to stop the disease deterioration in the late phase. Therefore, it is essential to track small changes in disease severity during daily life and take effective action to slow down the progression of PD. A personalized disease management plan is considered to be an effective approach for addressing the highly heterogeneous preferences and practical requirements of individual patients.

Wearable devices are important tools for personalized PD management. In the past decade, wearable devices have developed rapidly, thanks to the advances in communication technology and the Internet of Things (IoT). Remote monitoring of the patient’s daily lives with wearable sensing technology can assist healthcare professionals to gain insights into patients’ health conditions and empower PD patients to improve treatment effectiveness and slow disease progression (Dorsey and Eric, 2020). Mobile computing devices (e.g., smartwatches) have expanded the use of “on-body” applications from clinical settings to daily life based on body sensor networks (BSN).

Wrist-worn digital devices, especially smartwatches, are currently the most popular smart consumer wearable tools for healthcare diagnosis and self-management due to their convenience for long-term monitoring (Chakrabarti et al., 2022). In 2021, a methodological review surveyed the electronic health (eHealth) technologies for PD detection in daily life from the past two decades, while the management of symptoms was not investigated (Zhang et al., 2021). In 2022, a five-decade review outlined the progress of digital technology and computational techniques applied to PD motor symptom monitoring (Chandrabhatla et al., 2022). Non-motor manifestations of PD, such as sleep disorders and depression, can significantly decrease patient quality of life (QoL), yet are overlooked in this review. A recent systematic review highlights the deployed sensorial and algorithmic aspects of PD diagnosis and management (Giannakopoulou et al., 2022). These reviews do not consider the effect of the controlled environment, such as a clinic or laboratory on the clinical use of wearable devices in PD patients. Some activities of daily living (ADLs) mainly occur at home or home-like setting, and sleep quality is difficult to measure longitudinally in a clinic or lab. It is therefore essential to explore the clinical application of commercial wrist-worn devices for in-home PD monitoring. Wrist-based sensors have advantages such as convenience, wear compliance, and relative availability in the commercial market. There is a lack of review on the use of smartwatches for health telemonitoring from public databases, while a survey shows that most PD-related research concerns self-management in a laboratory environment (King and Majid, 2018). A recent review investigates whether the smartwatch can replace the role of a Parkinson’s disease doctor and concludes that the immediate implications for patients and clinicians are limited (Bloem et al., 2023). Remote digital monitoring of PD can become an important development for disease management and care.

Although the telemonitoring of PD using wrist-worn sensing has attracted significant interest in the research community, its value in daily PD management is still unclear. In this narrative review, we investigate the potential role of wrist-worn devices in PD motor and non-motor symptom monitoring in a natural, free-living environment. We evaluate the current technological progress as well as discuss the potential challenges and important future research directions of wrist-based technology in managing PD better.

## 2. Materials and methods

### 2.1. Reference searching method

This narrative review aims to summarize the current state-of-art of using wrist-worn technologies for monitoring motor and non-motor signs of PD, the different sensor types used in studies, and the potential for these devices in daily practice for the remote management of PD. To gather sufficient information for this review, we search for peer-reviewed journal articles from PubMed, IEEE Xplore, Web of Science, and Google Scholar using AI-based software ASReview LAB in October 2022. The search includes keywords: (“Parkinson” OR “PD” OR “Parkinson’s disease”) AND (“daily” OR “daily life” OR “home” OR “daily living”) AND (“wrist” OR “wrist-worn” OR “smartwatch” OR “wristband”).

### 2.2. Article inclusion and exclusion criteria

We select the articles based on the following inclusion criteria: (1) studies that focus on the use of wrist-based sensing technology for monitoring the motor or non-motor signs of PD, and (2) studies that are conducted in daily life or at home. Articles are excluded if (1) patients under study are not diagnosed with PD by a neurologist, (2) the article is not written in English, (3) the experiment is set up in the hospital or laboratory, (4) the device is unwearable or difficult to daily use at home, such as the wrist exoskeleton, or (5) there is no sensor in the monitoring device.

### 2.3. Information extraction

We extract four categories of data from selected articles: (1) Basic article information, including the authors and year of publication. We arrange the sources in chronological order to analyse the recent trend of published papers on wrist-based technology in PD monitoring. (2) The number of participants under study, including the number of PD patients and healthy controls, which are included to compare the credibility of different research. (3) Demographic information on the study population. To analyse the mild, moderate, and late-stage subgroups, we extract the disease duration of PD patients. To present the trend change in symptoms of PD, we also show the Hoehn and Yahr (H-Y) stage of PD patients (Goetz et al., 2004). To investigate the effect of total daily medication on patients’ daily symptoms, we extract the L-dopa equivalent daily dose (LEDD) (Julien et al., 2021). (4) Technical information: to differentiate the technical characteristic of commercial wrist-worn devices in references, we extract the outcome measures, location of the worn wrist, features, performances of wearable devices, sensor types, and clinical applications in the diagnosis and management of PD.

## 3. Results

### 3.1. Article selection and publication year

**FIGURE 1:**
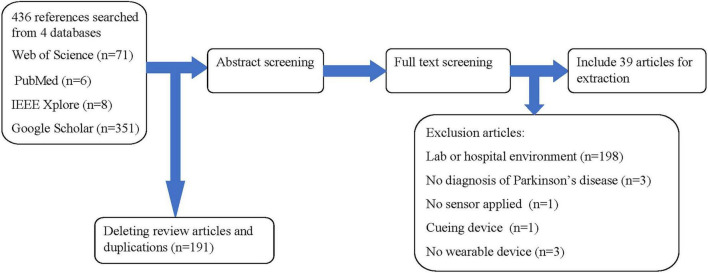
Reference selection and inclusion/exclusion procedure for this review.

**FIGURE 2:**
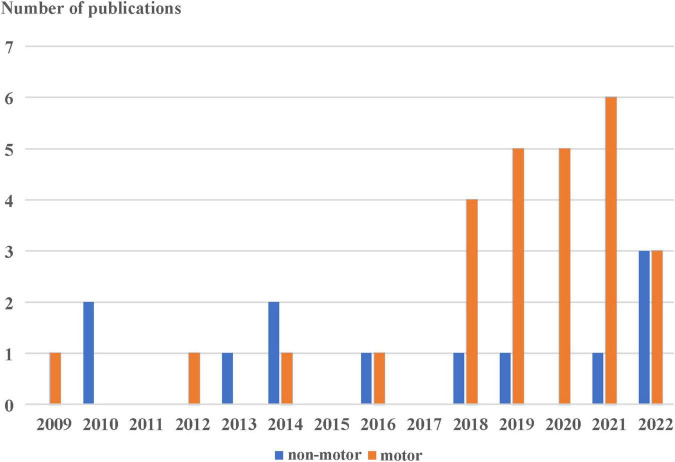
Published number of the study using the wrist-worn wearable in the monitoring of motor (orange) and non-motor (blue) symptoms of Parkinson’s disease.

We find 436 articles by searching the keywords. The articles come from a variety of sources: 71 articles from the Web of Science, 6 from PubMed, 8 from IEEE Xplore, and 351 from Google Scholar. After deleting review and duplicated articles through the use of ENDNOTE 20 software, 39 are selected for review according to our inclusion and exclusion criteria as can be shown in Figure 1. In Figure 2, the publication year is presented. The number of publications about commercial wrist-worn wearable devices applied in PD evaluation has increased significantly in the past 4 years, i.e., from 2018 to 2022; there are 29 articles published, accounting for 74% of the total studies considered.

### 3.2. Demographic and clinical characteristics

Table 1 provides information on the publication year, authors, number of participants, and demographics of the included studies. These studies use different research protocols with different numbers of patients and/or healthy controls. There are 14 case-control studies and 25 case studies. Most of the studies include a small number of patients (less than 100), with the exception of two papers with 304 and 388 participants, respectively. The participants in most of these studies are in the mild to moderate stages of PD, and only one study investigated the motor function of participants in the late stage of PD.

**TABLE 1:** Included articles and related information.

References	First author	Cases and controls	Disease duration in years (mean ± standard deviation)	H-Y stage	LEDD (mg/day)
Binder et al., 2009	Sabine Binder	10	5.6 ± 4.4	1	0
Naismith et al., 2010	Sharon L. Naismith	30/14	NA	NA	NA
Stavitsky et al., 2010	Stavitsky K.	22	NA	NA	NA
Roland et al., 2012	Kaitlyn P. Roland	15/15	NA	NA	NA
Bolitho et al., 2013	Samuel J. Bolitho	85/21	5.9 ± 5.2	2.0 ± 0.7	641.9 ± 466.3
Louter et al., 2014	Maartje Louter	45	9.5 ± 6.4	2.5	1089.4 ± 582.9
Gunn et al., 2014	David G. Gunn	95/48	5.3 (5.5)	2.0 (0.7)	594.5 (489.4)
Pulliam et al., 2014	Christopher L. Pulliam	15	3.5–17	NA	75–1930
Ossig et al., 2016	Christiana Ossig	24	NA	NA	NA
Klingelhoefer et al., 2016	Klingelhoefer L.	60	NA	NA	NA
Wu et al., 2018	Jade Q. Wu	35	NA	NA	NA
Pulliam et al., 2018	Christopher L. Pulliam	13	NA	2.6 (0.6)	1367 (768)
Rosqvist et al., 2018	Kristina Rosqvist	30	NA	4–5	799 (536–973)
Porta et al., 2018	Micaela Porta	18	9.9 ± 6.0	1.9 ± 0.4	NA
Silva de Lima et al., 2018	Ana Lígia Silva de Lima	304	NA	NA	NA
Isaacson et al., 2019	Stuart H. Isaacson	19/20	NA	NA	NA
Lang et al., 2019	Muriel Lang	30	11 ± 5	3.5	NA
Pradhan and Valerie, 2019	Sujata Pradhan	30/30	7.8 (5.0)	1.5	NA
Kim et al., 2019	Dong Wook Kim	46	7.6 (6.8)	2.2 (0.6)	NA
Hssayeni et al., 2019	Murtadha D. Hssayeni	19	9.2 ± 3.8	NA	1282.5 ± 459.8
van Wamelen et al., 2019	Daniel J. van Wamelen	108	7.5 (5.5)	2.9 (1.0)	950.4 (673.8)
Shah et al., 2020	Vrutangkumar V. Shah	29/27	NA	NA	NA
Abrami et al., 2020	Avner Abrami	25	NA	NA	NA
Pfister et al., 2020	Franz M. J. Pfister	30	NA	NA	NA
San-Segundo et al., 2020	Rubén San-Segundo	10	NA	NA	NA
Knudson et al., 2020	Mei Knudson	34	5.03 (1.40)	2.24 (0.43)	NA
Elzinga et al., 2021	Willem O. Elzinga	12	NA	1–3	NA
Kyritsis, 2021	Konstantinos Kyritsis	13/7	NA	NA	NA
Raykov et al., 2021	Yordan P. Raykov	25/25	NA	NA	NA
Tong et al., 2021	Lina Tong	5/5	NA	NA	NA
Habets et al., 2021	Jeroen G. V. Habets	20	8.1 (3.5)	NA	959 (314)
Sigcha, 2021	Luis Sigcha	18	NA	2.0 (0.78)	NA
van Wamelen D. et al., 2021	Daniel J. van Wamelen	12	NA	NA	NA
Ko et al., 2022	Yi-Feng Ko	27/30	NA	2–4	NA
Prusynski, 2022	Rachel A. Prusynski	25/27	NA	NA	NA
Raschellà et al., 2022	Flavio Raschellà	26/18	7.4 ± 5.9	2.0 ± 0.4	589.7 ± 275.6
Liu et al., 2022	Sen Liu	20	NA	NA	NA
Brand, 2022	Yonatan E. Brand	18/12	5.56 ± 4.05	2.3 ± 0.8	NA
Burq et al., 2022	Maximilien Burq	388	2.9 (1.4)	2.0 (0.5)	NA

H-Y stage, Hoehn and Yahr stage; LEDD, levodopa equivalent daily dose; NA, not applicable.

### 3.3. Motor symptoms monitoring

Between 2009 and 2017, we only find four publications focusing on the application of wrist technology for monitoring motor signs in PD. Figure 2 shows a strong increase in the use of wrist-based technology for monitoring PD motor signs in daily life, starting in 2018. The number of published papers on this topic increase by 4.5 times in the past 4 years, from 4 to 22. The majority of the studies (67%) focuses on motor symptoms of PD including rest tremor, bradykinesia, and gait.

Table 2 presents 26 studies that investigate the use of wrist-based wearable sensor devices for monitoring the motor features of PD patients over the past decade. These devices are typically worn for a mean of 6–7 days. To reduce the effect of motor symptoms on wearables’ sensitivity and specificity, the sensor is worn on the non-dominant wrist of patients. The motor commonly studied is tremor, which accounts for 34.6% (9/26) of all reviewed articles.

**TABLE 2:** Articles about wrist-worn commercial devices for telemonitoring motor signs in PD and related information.

References	Sensor type	Features	Performance	Clinical application	Measured outcome	Monitored duration	Wrist	Commercial name
Binder et al., 2009	Uniaxial accelerometer	Tremor duration and amplitude	Sensitivity	Assess tremor occurrence and severity	Tremor	6 weeks	Non-dominant	Actiwatch
Roland et al., 2012	GPS	Step counts, light physical activity time, sedentary time	NA	Examine gross mobility, assess PA, categorize stages of frailty	Frailty severity, PA	8 h	NA	Garmin Forerunner 405 GPS watch
Pulliam et al., 2014	Triaxial gyroscope and triaxial accelerometer	Acceleration and angular velocity	Average clinician total mAIMS scores and model scores	Quantify dyskinesia during unconstrained activities	Dyskinesia	10 days	bilateral	KinetiSense
Ossig et al., 2016	Triaxial accelerometer	Median BKS and DKS	NA	Capture motor fluctuations in patients with advanced PD	Motor fluctuations	5 days	Dominant	Parkinson’s KinetiGraph™ (PKG)
Pulliam et al., 2018	Triaxial gyroscope and triaxial accelerometer	Movement velocity, frequency	Sensitivity and specificity	Quantify the dose-response of rest tremor, bradykinesia, and dyskinesia in individuals with PD	Motor fluctuations	2 h	NA	Kinesia motion sensor units
Rosqvist et al., 2018	Triaxial accelerometer	Median BKS and DKS	NA	Continuous assessment of motor function	Motor fluctuations	10 days	NA	Parkinson’s KinetiGraph™ (PKG)
Porta et al., 2018	Triaxial accelerometer	Amount/intensity of PA, spatiotemporal and kinematic parameters of gait	Intensity	Predict possible changes in the gait pattern and verify the effectiveness of rehabilitative treatments and PA programs	PA and Gait	3 months	Non-dominant	ActiGraph
Silva de Lima et al., 2018	Triaxial accelerometer	Gait episode, time and frequency	Accuracy	Quantify walking quantity	Walking	10 h	NA	NA
Isaacson et al., 2019	Triaxial gyroscope and triaxial accelerometer	Tremor score, finger tapping speed score, DKS	Efficacy and safety	Provide feedback to patients on motor symptoms and supplement standard care to titrate the optimal rotigotine dosage.	Tremor, slowness, dyskinesia, and walking	12 weeks	NA	Kinesia 360
Lang et al., 2019	Triaxial gyroscope and triaxial accelerometer	Standard deviation, norm, maximum, root mean square, kurtosis, and skewness	Sensitivity and accuracy	Autonomous severity estimation of PD states	Tremor, dyskinesia, bradykinesia	331.2 ± 192.6 min	dominant	Schon Klinik Munchen Schwabing
Pradhan and Valerie, 2019	Triaxial accelerometer	Step counts and heart rate	User experience	Quantify the quantity and intensity of PA, provide feedback regarding activity levels	PA level	14 days and 14 nights	NA	Fitbit Charge HR (FBHR)
Kim et al., 2019	Triaxial accelerometer	Steps per day; activity counts per day; percent time spent sedentary, per cent time spent in light	NA	Estimate the motor activity	PA	1 week	non-dominant	ActiGraph
Hssayeni et al., 2019	Triaxial gyroscope and triaxial accelerometer	The peak-to-peak, dominant frequency	Specificity, sensitivity and accuracy	Assessment of response to medication	Motor fluctuations	6 months	Dominant	KinetiSense
Shah et al., 2020	Triaxial accelerometer, triaxial gyroscope, and triaxial magnetometer	The orientation and position trajectory of each foot (turn angle, swing time variability, etc.)	True/false positive fraction	Digital biomarkers of daily life mobility in PD	Mobility	1 week	Optional	Opals by APDM
Abrami et al., 2020	Triaxial accelerometer	Movement syllables	NA	Estimate changes in the PD state	Bradykinesia, tremor and PIGD	36 days	Non-dominant	GeneActive device (Activinsights)
Pfister et al., 2020	Triaxial gyroscope and triaxial accelerometer	Statistical features of the motion data	Feasibility	Detect the motor state	Motor fluctuation (off, on, dyskinetic)	NA	NA	NA
Knudson et al., 2020	Triaxial accelerometer	Median BKS and DKS	NA	Measure motor symptoms, predict activities of daily living impairment	Bradykinesia and dyskinesia	6 days	NA	Parkinson’s KinetiGraph™ (PKG)
San-Segundo et al., 2020	Triaxial accelerometer	The mean, range, or cross-correlation; the dominant frequency, energy content in a particular band, or signal entropy	AUC and FPR	Tremor detection, predict patient self-report measures	Tremor	4 weeks	Both	Axivity AX3
Raykov et al., 2021	Triaxial accelerometer	The step time, swing time, stance time, and double support time	Sensitivity and specificity	Detect gait and predict medication-induced fluctuations in PD patients based on free-living gait	Gait	At least 1 h	Both	Physilog 4
Tong et al., 2021	Triaxial accelerometer, triaxial gyroscope, and triaxial magnetometer	Root mean square value, variance, absolute mean, mean power frequency, peak power	Accuracy, sensitivity, and specificity	Hand tremor detection	Tremor	NA	Gominant	NA
Habets et al., 2021	Triaxial accelerometer	Extreme values, variances, jerkiness, number of peaks, and root mean squares; spectral power in specific frequency ranges; dominant frequencies	User-friendliness and feasibility	Classify the medication-induced fluctuations in bradykinesia	Bradykinesia	1 h	Unilateral	Physilog 4
Sigcha, 2021	Triaxial accelerometer	The amplitude and constancy of resting tremor	NA	Provide accurate and relevant information about tremor in patients in the early stages of the disease	Tremor	NA	Dominant	LDS V406 CE M4
van Wamelen D. et al., 2021	Triaxial accelerometer	Median BKS and DKS	NA	Capture bradykinesia scores of patients with *de novo* PD in a home setting	Bradykinesia	6 days	NA	Parkinson’s KinetiGraph™ (PKG)
Liu et al., 2022	Triaxial accelerometer	Dominant frequency, power dispersion, maxBin, mexBin, Kurt, Skew, SampEn	Accuracy, sensitivity, precision, and specificity	Record the long-term acceleration signals of PD patients with different tremor severities	Rest tremor severity	At least 2 h	Both	NA
Brand, 2022	Triaxial accelerometer	Rhythm, magnitude, regularity/consistency	Accuracy, precision, sensitivity, and specificity	Quantify daily living gait	gGait	10 days	Left	ActiGraph GT3X +
Burq et al., 2022	Triaxial gyroscope and triaxial accelerometer, PPG, and skin conductance sensors	NA	Sensitivity and reliability	Real-life distribution of disease severity	Tremor, bradykinesia, and gait	390 days	Dominant	Verily Study Watch

NA, not applicable; PA, physical activity; PD, Parkinson’s disease; GPS. Global Positioning System; BKS, bradykinesia score; DKS, dyskinesia score; PIGD, Postural instability and gait disorders; mAIMS, modified Abnormal Involuntary Movement Scale; AUC, area under curve; FPR, false positive rate; PPG, photoplethysmography.

**FIGURE 3:**
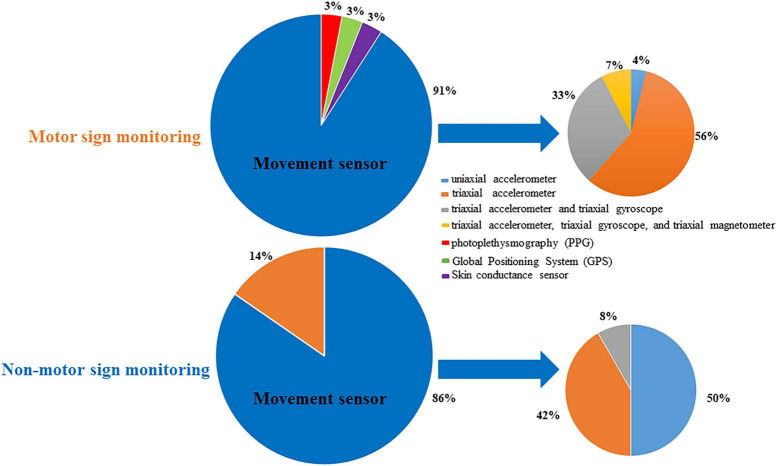
Pie chart showing the percentage of sensor types in PD signs monitoring devices.

Various research groups have employed different types of sensors in their research protocols. The pie chart (Figure 3) above shows that the most commonly used sensor type for motor signs monitoring is the movement sensor (91%). Interestingly, the triaxial accelerometer is the most frequently used movement sensor (60%). The gyroscope is another type of motion sensor that is frequently used to measure orientation and angular velocity.

**FIGURE 4:**
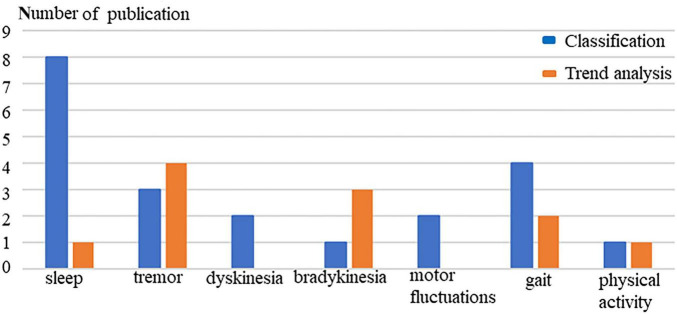
The technological application of wrist-worn sensors in monitoring PD symptoms. Classification (blue): Prediction and detection of symptoms; Trend analysis (orange): Progression or change of severity.

Figure 4 shows that the technical applications of wrist-worn sensors for monitoring rest tremor in PD include tracking tremor severity (*n* = 4) and detecting tremor signs (*n* = 3). In 2021, bradykinesia was first unobtrusively monitored by a wrist-worn accelerometer which indicated monitoring bradykinesia in non-invasive and non-intrusive ways to track the severity and progression of the symptom (Habets et al., 2021). Dyskinesia was first quantified in 2014 when the KinetiSense were worn on the bilateral wrists of PD patients during uncontrolled activities (Pulliam et al., 2014). Australian researchers developed the Personal KinetiGraph^®^ (PKG^®^) (Global Kinetics Corporation Ltd.) to produce the median bradykinesia score (BKS) and dyskinesia score (DKS) for objectively estimating the bradykinesia and dyskinesia of PD. Table 2 shows that the United Kingdom and Netherlands research group uses PKG to early detect bradykinesia in newly diagnosed and previously untreated PD patients (van Wamelen D. et al., 2021). Swedish and German research groups use it to assess the motor fluctuation of advanced PD patients and aim to guide the self-management of PD patients (Ossig et al., 2016; Rosqvist et al., 2018).

### 3.4. Non-motor symptoms monitoring

Table 3 shows 13 articles that have investigated non-motor symptoms (NMS) of PD in daily life with wrist-worn technology. The non-motor symptom monitors are mainly commercial actigraphy, including Actiwatch (*n* = 6), PKG (*n* = 2), ASUS VivoWatch BP (*n* = 1), Fitbit Charge HR (*n* = 1), and GENEActiv™ (*n* = 1). The actigraphy typically is worn on the non-dominant wrist. The reason is that the non-dominant wrist has fewer movements than the dominant one. Clearer data can be obtained when there are fewer movement artefacts. Therefore, PD patients can expect that the data can be acquired with fewer noises by wrist-worn on the non-dominant wrist. The duration of monitoring in these studies is typically 1 to 2 weeks. However, one study detects abnormal sleep phenomena in PD patients with a right wrist-worn device (ASUS VivoWatch BP) for 2 years (Ko et al., 2022).

**TABLE 3:** Articles about wrist-worn commercial devices for telemonitoring non-motor signs in PD and related information.

References	Sensor type	Features	Performance	Clinical application	Measured outcome	Monitored duration	Wrist	Commercial name
Naismith et al., 2010	Uniaxial accelerometer	Rest interval onset and offset, rest efficiency, wake bouts	Utility	Early identification of RSBD and guide early intervention	Sleep	2 weeks	Non-dominant	Actiwatch
Stavitsky et al., 2010	Uniaxial accelerometer	Sleep onset latency, sleep efficiency, wake after sleep onset, total sleep time, sleep fragmentation	Utility	Measure sleep quality	Sleep	7 days and nights	Each	Actiwatch
Bolitho et al., 2013	Uniaxial accelerometer	Duration and correlates of excessive daytime napping	NA	Objective measure of daytime sleep	Sleepiness and cognition	2 weeks	Non-dominant	Actiwatch
Louter et al., 2014	Uniaxial accelerometer	Total sleep time, sleep efficiency, sleep latency, no wake bouts, length wake bouts, activity score	Sensitivity, specificity and positive predictive value	A diagnostic aid for RSBD in Parkinson’s disease	Sleep	7 nights	Non-dominant	Actiwatch
Gunn et al., 2014	Uniaxial accelerometer	Sleep efficiency, sleep onset/offset (variability)	NA	Assessment of sleep disturbance	Sleep and cognition	2 weeks	Non-dominant	Actiwatch
Klingelhoefer et al., 2016	Triaxial accelerometer	Parameters for sleep quality and quantity	NA	Objective remote marker of disturbed nighttime sleep	Sleep	6 days and nights	NA	Parkinson’s KinetiGraph™ (PKG)
Wu et al., 2018	Uniaxial accelerometer	Total sleep time, sleep onset latency, wake after sleep onset, and sleep efficiency	Utility	Rest-activity rhythm as a biomarker for circadian function in PD	Cognition	7−10 days	Non-dominant	Actiwatch
van Wamelen et al., 2019	Triaxial accelerometer	Mean BKS and DKS	NA	Measure the non-motor symptoms correlate with BKS and DKS	9 domains of non-motor symptoms	6 days	Dominant	Parkinson’s KinetiGraph™ (PKG)
Elzinga et al., 2021	PPG	HR and sleep state estimates	Repeatability and Minimum Detectable Effect	Detect clenbuterol-induced changes and track treatment effects	Sleep	6 days	NA	NA
Kyritsis, 2021	Triaxial gyroscope and triaxial accelerometer	Bite moments and upwards wrist micro-movements	Sensitivity and specificity	Classification of in-meal eating profiles to the PD or the healthy populations	Gastrointestinal	7 days	Dominant	NA
Ko et al., 2022	Triaxial accelerometer, PPG	Sleep efficiency, REM, and sleep cycle	Accuracy	Detect the abnormal RBD phenomenon	Sleep	2 years	Right	ASUS VivoWatch BP
Prusynski, 2022	Triaxial accelerometer	Nighttime sleep, wakenings after sleep onset, number of wakenings, naps, step count, and PA intensity	Intensity	Measure the sleep and sedentary behavior in mild PD	Sleep	2 weeks	NA	Fitbit Charge HR
Raschellà et al., 2022	Triaxial accelerometer	Individual movement episodes, global nocturnal activity	Accuracy, sensitivity, and specificity	Automatic RBD diagnoses in home settings	Sleep	2 weeks	NA	GENEActiv™

NA, not applicable; PPG, photoplethysmography; HR, heart rate; REM, rapid eye movement; RSBD, rapid eye movement sleep behavior disorder; RBD, REM behavior disorder; BKS, bradykinesia score; DKS, dyskinesia score.

Most NMS studies (8 out of 13) focus on sleep quality. Interestingly, one article classifies gastrointestinal symptoms in PD using Inertial Measurement Units (IMUs) (Kyritsis, 2021). Figure 3 shows that sensor types used to measure NMS include movement sensors (*n* = 12, 86%) and photoplethysmography (*n* = 2, 14%). The uniaxial accelerometer is the most frequently employed movement sensor (50%).

## 4. Discussion and conclusion

This review is the first to provide an overview of the use of wrist-worn devices for remote monitoring and managing PD patients in their daily living environment. Compared with the classification of PD symptoms, wrist-worn sensors have been less commonly used to daily track the progression of PD. In the last decade, researchers have primarily used movement sensors, i.e., accelerometers, to daily monitor motor and non-motor symptoms of PD.

### 4.1. Symptoms monitoring

In-home monitoring using wrist-worn technology has primarily focused on classifying and managing motor symptoms and sleep disorders of PD patients, leaving many non-motor symptoms unaddressed. With the development of digital mobile technology, technology-based objective measurements, particularly wrist-worn monitors have become popular among patients and clinicians in daily monitoring (King and Majid, 2018). Researchers have mainly used mobile devices to help PD patients detect, monitor, and manage motor symptoms while neglecting non-motor symptoms which are difficult to measure directly by wearable sensors every day. This is reflected in the fact that 67% of the studies reviewed focus on motor signs, compared to only 33% that focus on non-motor signs. Non-motor symptoms remain a neglected area of research in PD monitoring.

Previous works on PD motor monitoring mainly focused on three cardinal signs: tremor, dyskinesia, and bradykinesia. The wrist joint is most frequently affected by rigidity. Table 2 shows that there is no study on rigidity monitoring with the wrist-based sensor in a daily environment. A recent scoping review of wrist rigidity evaluation reported that some force and inertial sensors have been used to quantitatively assess the rigidity of wrists in PD (Alves et al., 2022). However, it is challenging to standardize the measurement of rigidity with accessible wearables. With the fast development of technology and computational techniques, it is likely that new analytical models for sensor application in rigidity emerge in the future.

Compared with 26 studies focusing on motor signs, only half of them have investigated the non-motor signs in daily life. In particular, sleep problems have been the focus of PD research. Rapid eye movement sleep behavior disorder is thought to be a prodromal symptom of PD and has been targeted for disease-modifying treatment (Naismith et al., 2010). Actiwatch is most frequently used to quantify sleep features, such as sleep efficiency, and sleep onset/offset (Naismith et al., 2010; Stavitsky et al., 2010; Bolitho et al., 2013; Gunn et al., 2014; Louter et al., 2014). Daniel et al. used PKG to measure the correlation between non-motor symptoms and bradykinesia and dyskinesia scores in PD patients. They suggested that the future of digital technologies may enable the reliable measurement of often under-reported and under-recognized non-motor signs (van Wamelen et al., 2019; van Wamelen D. J. et al., 2021). In addition to motor problems, non-motor symptoms impact 90% of PD patients. Some subjective non-motor symptoms, such as fatigue and depression, can significantly decrease the quality of life of PD patients and are outside the focus of Parkinson’s care and research. These non-motor problems are highly variable and present throughout the disease’s progress. The gap between these unmet needs of non-motor endpoints and technology platforms is currently large. New sensor technology and computational models, such as deep learning and semi-supervised learning may be applied to smart wrist-worn devices to improve the management of non-motor symptoms in PD.

### 4.2. Wearable healthcare sensor

Compared to traditional scales, body-worn sensors (BWS) record the data of patients’ symptoms in a long-term, real-time, and objective manner. Additionally, BWS may reduce the cost of time and money for patients seeking professional care. A large amount of data collected by BWS may be valuable for monitoring symptoms. BWS can be worn on the wrist, waist, or ankle, and provide PD patients and healthcare professionals with relevant information in daily-living conditions. Compared to locations of the body, the wrist-worn sensor is easy to view and simple to operate in daily life by older individuals.

Different kinds of sensor types have different functions in clinical applications of PD monitoring. The accelerometer is the most frequently used wrist-worn sensor in monitoring PD motor and non-motor signs in the natural living environment. Among motion sensors, the triaxial accelerometer most frequently detects motor signs of PD, while the uniaxial accelerometer is most frequently used for monitoring sleep signs. The possible reason is that a single triaxial accelerometer can classify the signals of basic daily movements in a PD patient’s activity, while the uniaxial accelerometer only classifies postural orientations during rest. In addition to movement sensors, photoplethysmography (PPG) sensors are also frequently used in monitoring non-motor and motor signs (Elzinga et al., 2021; Burq et al., 2022; Ko et al., 2022). PPG is a simple and low-cost wearable device for monitoring blood flow and blood oxygenation. Wrist PPG signal is widely used in heart rate monitors. The role of PPG in PD clinical practice still has a lot of potential. However, a single sensor is probably insufficient to detect all relevant signs of the body in PD patients. Accelerometers and other sensors do not offer sufficient and practical data for the real-time assessment of motor signals. Global Positioning System (GPS) sensors do not penetrate solid walls and are affected by large structures, which means that patients cannot use GPS indoors or in the underground environment. In the future, researchers should design multimodal measurements to monitor different clinical manifestations of PD. IMU and other multimodal sensors can help capture more clinically meaningful signs in daily monitoring and are expected to better monitor PD manifestation than a single-modal sensor. For example, Maximilien et al. studied motion sensors connected with skin conductance sensors and PPG to capture multimodal data of PD for standard virtual motor exams (Burq et al., 2022). Meanwhile, it is important to balance the amount of information being collected with the number of sensors when developing a standard technology measurement platform. Also, it is important that these data systems have high standards for patient data security and privacy.

### 4.3. Value in the daily management

Wrist-worn sensors might have the potential to help clinicians and patients to detect PD symptoms at an early stage through increasing the awareness of patients and offering clinicians deep insight into patients’ daily life situations, predict the motor and non-motor signs and subsequently track PD symptom severity in daily naturalistic environments. From Figure 4, we find that classification of wrist-based technology is mainly in sleep, gait, and tremor of PD, because wrist-worn accelerometers can easily classify activity patterns in daily living. Moreover, wrist-worn devices have been widely used by the public with the popularity of smartwatches. The common usage of the devices can timely offer healthcare professionals opportunities to detect PD symptoms in the high-risk population at an early stage. Wrist wearables mainly analyse the trend of rest tremor and bradykinesia, but have not been used very often to track NMS. The lack of proper sensing technology may be the cause of the gap which will need to be addressed in future studies. In addition, wrist-worn devices might be helpful in the daily management of PD patients, but further research must be done as the correlations so far with clinical scales are not very high.

In line with our findings, a recent in-depth analysis based on over 50 articles supported that little focus has been placed on the management of PD NMS *via* wearable sensors, compared to much work that has been attributed to PD motor symptom management (Mughal et al., 2022). Wrist-worn devices can be used to detect prodromal non-motor signs in daily life, such as sleep disorders, which can serve as digital biomarkers in the prodromal phase of PD. Actigraphy can be used to measure abnormal sleep-related features in daily life and guide portable interventions for precision medicine in patients with specific phenotypes. In 2022, a review reported that lifestyle intervention is the first test to prevent PD (Janssen Daalen et al., 2022). Digital remote devices can provide exercise interventions to prodromal patients in daily life, monitored by experts, which may also help early detection of specific prodromal NMS, objectively measure the outcome of disease progression, and guide long-term self-management. However, the NMS is a field which is still to be explored. There is no device certified under the United States Food and Drug Administration (FDA) or European Medicines Agency (EMA), so articles found so far only explore the possibility to use them, and NMS device development and validation are still needed to be done in future.

Furthermore, if the phenotype of patients is better defined, diagnosing and managing the complex disease can be more accurate. Currently, late-stage patients are often ignored, and more advanced sensor technology is needed to address their specific needs. Early diagnosis of PD before clinic visits and the use of sensor technology for self-management at home are two areas where there is potential for clinical applications of sensor technology for PD.

### 4.4. Current pitfalls in using wrist-worn devices in the clinical PD management

Considering the convenience of smartwatches in daily practice, we propose to use wrist-worn monitoring in health self-management. However, there are also some limitations that may hinder the wrist-worn monitoring technology to be translated into PD clinical management in daily life. (1) One of the most important pitfalls is the lack of standardization of different wrist-based objective measurements. For example, commercial wrist-worn devices have different measurement protocols and different feature extraction algorithms behind PD symptoms. The heterogeneity can hinder the standardization of clinical practice guidelines about using wrist-worn devices for PD management. In future, the obligation of certification by a recognized agency of medication, such as the FDA or EMA, can set standard technology assessment criteria for different competitors’ devices. (2) Another pitfall is about missing medical device certificates in most wrist-worn devices. The certification of the devices indicates the safe and reliable usage for customers to use it track the disease and manage their health. Before the clinical trials of wearable devices are completed, the technology cannot be approved by the professional authority, which becomes the main barrier to pushing forward at-home monitoring. Effective collaborations between private companies and academic initiatives can further advance this field. (3) The limitation of using wrist-worn movement sensors for the complete human body movement analysis is another pitfall for clinical PD management. Wrist technology has the limitation of motion data being detected only from the arm, rather than the whole body in the field of human movement science. Meanwhile, false negative and positives events should be considered in detecting some PD symptoms from the wrist location. For example, the wrist worn devices misses several movements in the rest of the body a part of missing the axial symptoms. In addition, the high degrees of freedom in the arm adds to the randomness of movements in the arms, provokes overestimation of several movements and false positives events (Gjoreski et al., 2016; Shcherbina et al., 2017). The current possible solution for accurate PD motor symptom monitoring is to use multiple sensors placed at different body parts, for example, combining a smartwatch with a smartphone, or using non-wearable sensor technologies such as wall-mounted devices that can non-invasively capture the body movement patterns. In many cases, the accuracy of the calculated variables is still poor, and the measurement accuracy of many wearables is even not validated for people with PD. To increase the accuracy of wrist-worn techniques in PD motor symptom monitoring, we should further develop the ambulatory human movement analysis field that aims to capture the whole body movement using the minimal number of movement sensors on the body. According to the current ambulatory human movement analysis techniques, we would recommend that PD researchers and healthcare professionals use waist- or chest-worn sensor to better characterize axial movements, such as, bradykinesia and dyskinesia and better model the gait patterns of human subjects than only using the wrist-worn sensor. (4) Researchers should also consider that PPG and electrodermal activity sensors are strongly influenced by the wearing of wrist-worn devices during non-motor symptom monitoring. The loose wearing of the devices can decrease the quality of signals given the technical mechanism of the sensors. However, wearing too tight can affect the usability of the device and then increase users’ dropout rate in using the devices for their PD management. It is essential for healthcare professionals to find a balance between the correct and comfortable usage of the wrist-worn device and provide their patients with some practical guidelines. Moreover, the relevant algorithms to extract physiological variables from PPG and electrodermal activity are still necessary to be validated according to the actual daily life usage of the wrist-worn devices for people with PD.

### 4.5. Limitation

There are a couple of limitations to this narrative review that should be noted. Firstly, this narrative review does not use systematic statistical methods to assess the quality of studies, and may make it less reproducible compared to a systematic review. Additionally, the review might contain selection and evaluation biases. Secondly, the review does not provide a systematic discussion about the data analysis techniques employed in the studies that are surveyed. The clear description of algorithms behind the extracted features enables healthcare professionals and patients to make decisions based on transparent information for PD disease management. However, we noticed that most clinical articles we reviewed did not describe the algorithms clearly. In addition, this review focuses on the potential value of investigating wrist technology in the management in daily life, and the feature extraction algorithm and monitoring performance are out of the scope of this review. Therefore, we did not include information about feature extraction algorithms and relevant monitoring performances in this review. We recommend that it is valuable to have a technique-oriented review discussing data analysis methods behind the usage of wrist-worn devices in PD management to contribute to the accuracy of PD symptom monitoring in daily life.

### 4.6. Conclusion

Wrist-worn monitoring technology with medical device certification has the potential to improve early intervention and personalized care management of PD patients based on daily health information exchange. Wrist-worn PPG sensor combined with motion sensors could assist to estimate motor symptoms, analyze daily activity, and even address some unmet needs for non-motor symptoms of PD patients in daily life. However, only a few studies have addressed the need of non-motor symptom monitoring among PD patients. More emphasis should be placed on non-motor symptom monitoring using wrist-worn devices in the future. In addition, clinical healthcare professionals and patients should pay attention to the shortcomings of commercialized wrist-worn devices, such as, the lack of reliability, sufficient quality, and clinical validation. More wrist-worn technology assessment and clinical validation studies are recommended in future to increase the technique trustworthiness and effectiveness in the clinical management of PD in daily life.

## Author contributions

PL made the draft of manuscript. PL and YW made the main design flow of the review. RW and YW gave the main comments to the manuscript. YZ and FH gave the grammatical and other revised suggestions to the draft. All authors approved the final revision to be published.
